# A Rare Case of Ovarian Filariasis in Abidjan

**DOI:** 10.1155/2016/4075162

**Published:** 2016-05-23

**Authors:** Doukouré Brahima, Abouna Alain Didier, Dou Gonat Serge Pacôme, Aman Nguiessan Alphonse, Koffi Abdoul, Diomandé Mohenou Isidore Jean-Marie

**Affiliations:** ^1^Anatomic and Cytological Pathology Department, Cocody Teaching Hospital, BP 368 Abidjan 08, Côte d'Ivoire; ^2^Parasitology and Mycology Department, Cocody Teaching Hospital, Côte d'Ivoire; ^3^Anatomic and Cytological Pathology Department, Bouake Teaching Hospital, Côte d'Ivoire; ^4^Obstetrics and Gynecology Department, Yopougon Teaching Hospital, Côte d'Ivoire

## Abstract

Ovarian filariasis is an exceptional disease and displays a major diagnostic problem even in endemic areas. We reported the case of a 19-year-old patient who had ovarian cyst which was revealed by chronic pelvic pain. The histological examination of oophorectomy specimen led to the* Wuchereria bancrofti* filariasis of the ovary. The anatomopathologic examination is required for the diagnosis of this disease.

## 1. Introduction

Lymphatic filariasis is a parasitic major endemic disease due to round worms of the genera* Wuchereria* and* Brugia*. It roughly affected 120 million people in 73 tropical countries and represents the second leading cause of permanent disability worldwide according to the WHO [1]. In addition to the usual locations, it can be found at other organs. Pelvic or ovarian sites remain rare despite described cases in the literature [2, 3]. We report a case of ovarian localization of filariasis in a 19-year-old patient based on the literature review.

## 2. Observation

GD was 19 years old and nulliparous without any particular medical background. She presented with intermittent pelvic pain from more than 6 months. The history did not reveal any risk factor nor entry point. Clinical examination found an abdominal pain syndrome in the left iliac fossa. There was no hepatosplenomegaly and no alteration of general state.

The blood count showed a slight increase of eosinophilia, and the pelvic ultrasound has pinpointed a heterogeneous image characterizing an organic ovarian cyst.

The patient underwent cystectomy. Intraoperatively, a ruptured mixed ovarian cyst was observed. The sample was fixed in 10% formalin and sent to the Anatomic Pathology Laboratory for histological analysis.

Macroscopically, the ovary weighed 74 g, measured 10 cm × 07 cm × 05 cm with a regular smooth surface, and had both semisolid and semicystic consistency. The section of ovary showed a 3 cm cyst containing gelatinous substance without vegetation and a fleshy and a whitish area associated with haemorrhage.

**Figure 1:**
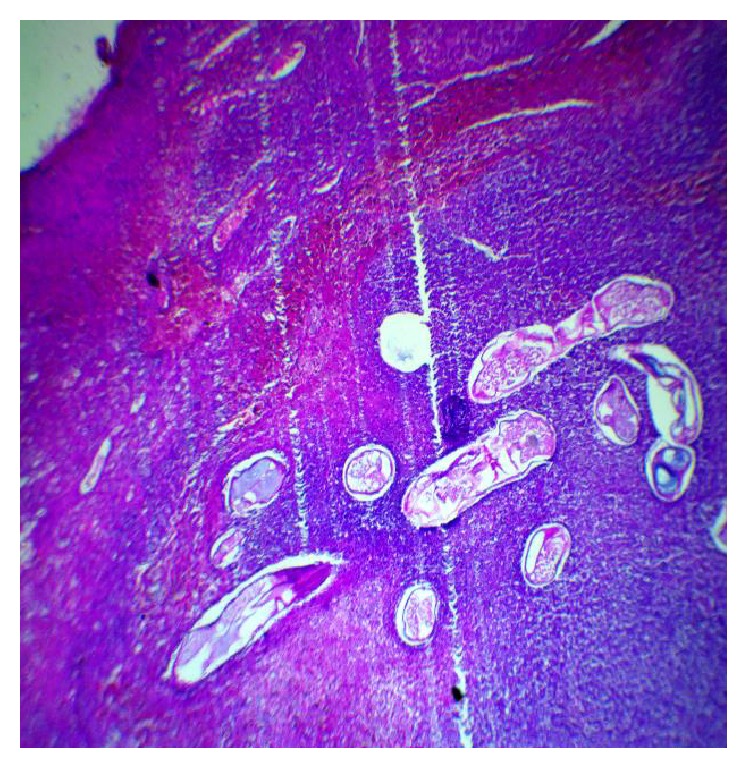
HE ×100: cuticle segments of adult worms containing microfilariae on a necrotico inflammatory and background.

At microscopical level, we found an infiltrate of polymorphonuclear leukocytes along with the presence of cuticle of adult worms containing microfilaria (Figure 1) suggesting the diagnosis of ovarian filariasis which was confirmed by direct parasitological examination (ovarian* Wuchereria bancrofti* filariasis).

The postoperative follow-up care is simple, and therefore, the patient is healthy.

## 3. Discussion

Lymphatic filariasis caused by* Wuchereria bancrofti* affects millions of people in tropical countries. This disease considerably causes severe disability and has significant socioeconomic implications.

*Wuchereria bancrofti*, an infectious agent of lymphatic filariasis in 95% of cases, is recognized as the second most disabling disease after malaria among the diseases transmitted by mosquitoes [1]. There are two forms of contamination which could be primary or secondary. The secondary form is the most common because of the presence of adult worms in the lymph system resulting in its obstruction [4, 5], while the primary form is rare. The primary form seems to be exceptional since after inoculation, the microfilariae have a centrifugal progression from the root to the tip of the members.* Wuchereria bancrofti*,* pacifica*, and* Brugia malayi* affected the lower limb, the upper limb, and the leg along with the ankle and the popliteal fossa, respectively [6]. Our observation seems to be a primitive localization of* Wuchereria bancrofti*, since we did not find any other site. Moreover, men are more affected than women. The ovarian localization of lymphatic filariasis is rare and only a few cases have been reported in the literature [2–5].

Additionally, the diagnosis of ovarian filariasis is rarely made in preoperative condition because clinical symptomatology simulates that of a functional cyst, and thus, the histologic examination is significant for the validation the ovarian filariasis diagnosis. Surgery and medical therapy primarily helps to avoid recurrences [1, 5]. In our case of study, the patient underwent an ovariectomy followed by cures of albendazole and ivermectin. The prognosis of this disease is usually favorable.

## 4. Conclusion

Ovarian filariasis is an exceptional disease even in endemic areas. Only the histopathological analysis of sampled tissues helps diagnose this disease. Postoperative surveillance should be systematic; however, prevention remains as the best treatment strategy plan for filariasis.
